# Measurements of the Hansen solubility parameters of mites and cockroaches to improve pest control applications

**DOI:** 10.1016/j.heliyon.2019.e01853

**Published:** 2019-06-08

**Authors:** Yuki Kato, Shinichi Tsutsumi, Nobuyuki Fujiwara, Hideki Yamamoto

**Affiliations:** Department of Chemical, Energy and Environmental Engineering, Faculty of Environmental and Urban Engineering, Kansai University, Japan

**Keywords:** Physical chemistry, Analytical chemistry, Hansen solubility parameter, Mites, Cockroaches

## Abstract

Various pests, such as cockroaches and mites, can negatively affect agriculture and human health. Many pesticides have been developed to control these pests. The surfaces of pests are hydrophobic, so an insecticide in an aqueous solution will be repelled by the surface of a pest and therefore will not be effective. Adding a spreading agent (e.g., a surfactant) will improve the ability of a pesticide solution to wet pest surfaces and therefore improve the ability of the active ingredient to permeate and kill pests. Efficiently killing insects requires the insecticidal component to have an affinity for the pest surface. This affinity was assessed here using the Hansen solubility parameter, which is a quantitative measure of the affinity between two substances. We determined HSPs of mites and cockroaches using Hansen solubility sphere method. The HSPs of mites were *δ*_*d*_ = 16.4 (MPa)^1/2^, *δ*_*p*_ = 2.6 (MPa)^1/2^, and *δ*_*h*_ = 4.7 (MPa)^1/2^. The one HSPs of cockroaches were *δ*_*d*_ = 15.5 (MPa)^1/2^, *δ*_*p*_ = 20.4 (MPa)^1/2^, *δ*_*h*_ = 20.2 (MPa)^1/2^, and others were *δ*_*d*_ = 17.6 (MPa)^1/2^, *δ*_*p*_ = 2.8 (MPa)^1/2^, and *δ*_*h*_ = 3.8 (MPa)^1/2^. The HSPs of cockroaches showed two values of hydrophobicity and hydrophilicity. Finally, we proposed new derived guidelines for using Hansen solubility parameters in research into pest control agents.

## 1 Introduction

Pests such as cockroaches and ticks are found throughout Japan. These pests can be found in kitchens, living rooms, and other parts of houses, and can produce allergens that cause bronchial asthma and allergic reactions in the eyes and nose. It has been found in epidemiological studies that the prevalence of bronchial asthma and allergic rhinitis in Japanese people older than 20 y are ∼5% and 30%–40%, respectively [1, 2]. In 1994, Elke et al. analyzed enormous amounts of data on eight economically important crops and concluded that “we are potentially producing 70% loss on the basis of production value”. This means that only 30% of the total yields could be produced if pests were not excluded [3].

An insecticide preparation for agricultural use will generally contain a surfactant to act as a spreading agent. The surface of an insect is hydrophobic, to protect the respiratory organs from rainwater, so an insecticide solution that does not contain a surfactant will be repelled by the surface of the insect, and the active ingredient will not be effective. Adding a surfactant (i.e., a substance with a hydrophobic component and a hydrophilic component) improves the ability of the solution to wet the insect surface, improving the efficiency with which the active ingredient can permeate and kill the insect. Effectively controlling insect pests requires the insecticide component to quickly permeate the body, so the affinity of the insect surface and the insecticide is important [4, 5, 6]. Hirashima et al. studied quantitative structure–activity relationships between grasshopper surfaces and octopaminergic agonists and antagonists using physicochemical parameters such as the octanol–water partition coefficient [7]. Studies of the effects of insecticides on the nerves of pests and of the repellencies of pest surfaces to insecticides have been performed [8, 9]. However, few empirical studies of the affinities of insecticides for insect surfaces to allow better insecticides to be developed have been performed. We have used the Hansen solubility parameter (HSP), which is used to evaluate the affinity between two substances, to quantify the properties of the surfaces of pests and the affinities between pest surfaces and pesticides [10, 11]. The HSP can quantitatively indicate the affinity of two substances, and has been used in research into food, gas, petroleum, and other materials in recent years [12, 13, 14]. Darija et al. used HSPs to assess the gastrointestinal sites relevant to atypical antipsychotics such as aripiprazole, and identified the aripiprazole decomposition products. The HSP is expected to be useful in biological studies [15].

The solubility parameter *δ* (in (J/cm^3^)^1/2^) is a physical property defined by Hildebrand as an indicator of the solubility of a substance. The HSP is defined as the solubility parameter *δ*_*t*_ ((MPa)^1/2^) divided into three terms, a dispersion force term, a polar forces term, and a hydrogen-bonding force term [11].

Three methods have been used to determine the HSP, the Hansen solubility sphere method [14], the molecular group contribution method [16], and a method involving calculations using correlations between physical properties and various parameters [11]. The Hansen solubility sphere method gives the HSP through an affinity evaluation taking interactions with various organic solvents into consideration. This method can be used regardless of the state of the sample. The molecular group contribution method involves applying parameters for various molecular groups and is suitable for calculating the HSP of a single component with a known structure. The physical property estimation method is used to estimate an HSP from correlation formulae for the relationships between various physical properties and the HSP, and is suitable for calculating the HSP of a liquid sample.

The aim of this study was to measure the HSPs of *Dermatophagoides* mites and German cockroaches to allow new guidelines for developing pest control agents to be proposed. Each pest was defined as a solid multicomponent system with various ingredients. We performed affinity evaluation tests using various organic solvents and determined the HSPs using Hansen solubility sphere method to attempt to quantitatively evaluate the physical properties of the pests. Based on the measured results of HSP, we selected substances that could be used as insecticides with good affinity to various pests. Finally, we proposed new development method for using HSPs in research into pest control agents.

## 2 Theory

### 2.1 Solubility parameter

The Hildebrand solubility parameter *δ* (in (MPa)^1/2^) is a value defined by Hildebrand as an index describing solubility expressed in terms of cohesive energy and molar volume. The Hildebrand solubility parameter is calculated using the equation

$$\delta ={\left(\frac{\Delta E}{V_{m}}\right)}^{1/2},$$

where *ΔE* and *V*_*m*_ are the cohesive energy (J) and molar volume (cm^3^/mol), respectively. The Hildebrand solubility parameter, which represents the cohesive energy density, has been used to evaluate the compatibilities and dispersibilities of materials. However, the Hildebrand solubility parameter is a nonspecific physical property parameter and does not distinguish between polar interactions, nonpolar interactions, and other interactions. So the affinity between two specific materials, such as alcohols, cannot be appropriately evaluated, and the parameter does not conform to theory.

To solve this problem, Hansen divided the Hildebrand solubility parameter *δ* into three components defined using the equations

$$\delta_{d}=\left(\frac{\Delta E_{d}}{V_{m}}\right),\;\delta_{p}=\left(\frac{\Delta E_{p}}{V_{m}}\right)\text{and}\;\delta_{h}=\left(\frac{\Delta E_{h}}{V_{m}}\right),$$

where

$$\delta_{t}={\left(\delta_{d}+\delta_{p}+\delta_{h}\right)}^{\frac{1}{2}},$$

in which *δ*_*d*_, *δ*_*p*_, and *δ*_*h*_ are dispersion force, polar force, and hydrogen-bonding force terms, respectively.

The dispersion force term *δ*_*d*_ describes general van der Waals interactions between substances. There will be a powerful attractive force between any two molecules a few Ångströms apart. These forces are everywhere, so tend to be ignored, but they dominate most interactions.

The polar force term *δ*_*p*_ describes the familiar “positive attracts negative” electrical attraction caused by dipole moments. These forces are important for almost every type of molecule except some hydrocarbons and certain molecules containing only carbon and fluorine.

The hydrogen-bonding force term *δ*_*h*_ describes forces that are arguably polar but whose predictive value goes beyond thinking of them as simply polar forces, so they are considered to be distinct. Hydrogen-bonding forces can generally be considered to describe electron exchange, so CO_2_ has strong “hydrogen-bonding” forces that make it a good solvent [11, 16].

Quantitatively evaluating the affinity between two substances can be achieved using *R*_*a*_ ((MPa)^1/2^), which reflects the distance between the HSPs of the two substances. *R*_*a*_ can be calculated using the equation

$$R_{a}={\left\{4{\left(\delta_{d1}-\delta_{d2}\right)}^{2}+{\left(\delta_{p1}-\delta_{p2}\right)}^{2}+{\left(\delta_{h1}-\delta_{h2}\right)}^{2}\right\}}^{\frac{1}{2}},$$

in which subscripts 1 and 2 indicate components 1 and 2, respectively [14].

A smaller *R*_*a*_ value will indicate that the HSPs for the two substances are more similar and the substances have more affinity for each other.

The HSP for a mixed solvent can be calculated using the equations shown below, taking the volume fraction of each substance into consideration [11].

$$\delta_{d,mixed}=\varphi_{1}\delta_{d,1}+\varphi_{2}\delta_{d,2}$$

$$\delta_{p,mixed}=\varphi_{1}\delta_{p,1}+\varphi_{2}\delta_{p,2}$$

$$\delta_{h,mixed}=\varphi_{1}\delta_{h,1}+\varphi_{2}\delta_{h,2}$$

In these equations, subscripts 1 and 2 indicate components 1 and 2, respectively.

### 2.2 Hansen solubility sphere method

A method for calculating HSPs from the results of affinity evaluation tests is described here. Initially, the affinities are evaluated using organic solvents with known HSPs. Affinity evaluation methods taking compatibility, swelling properties, and dispersibility are available. A method suitable for the sample of interest is selected. HSPs for a good solvent with a good affinity and a poor solvent with a poor affinity are plotted on a Hansen three-dimensional graph. The smallest radius sphere (Hansen sphere) is then created using the data for the good solvent within the sphere and the data for the poor solvent outside the sphere. The center coordinates of the sphere give the HSP for the target substance [17, 18].

The HSP of a substance will generally be a single value. That is, the Hansen solubility sphere gives one HSP. It has recently become clear that certain substances have two types of HSP (i.e., two types of Hansen sphere). Abbott et al. found that a surfactant (i.e., a substance with both hydrophobic and hydrophilic properties) will have a hydrophilic Hansen solubility sphere and a hydrophobic Hansen solubility sphere [19]. Agata et al. found that an ionic liquid (which will have a polar component and a nonpolar component) will also have two types of Hansen sphere [20]. In summary, substances with different components (e.g., surfactants and ionic liquids) will have two types of Hansen sphere, one related to each component. The HSPiP program has a program to create two kinds of Hansen solubility spheres.

### 2.3 Constituents of pests

Leopard mites are pests in the Acaricidae family. These mites inhabit dust in buildings, and can produce allergens that cause bronchial asthma and allergy symptoms in the eyes and nose. It has been presumed that the main components of leopard mite surfaces are cholesterol and squalene [21]. The approximate *δ* values for leopard mites were *δ*_*d*_ = 17.1 (MPa)^1/2^, *δ*_*p*_ = 1.5 (MPa)^1/2^, and *δ*_*h*_ = 3.5 (MPa)^1/2^. Organic solvents for the affinity evaluation were selected as described above.

German cockroaches are pests in the cockroach family. German cockroaches are found widely in buildings around Japan. Dead German cockroaches and German cockroach excreta produce fine particles that can become suspended in indoor air and cause bronchial asthma and similar problems [2]. The main component of German cockroach bodies is chitin (like for shrimps and crabs), and the body surfaces are covered with oil that prevents moisture entering the air channels [22]. Organic solvents for the affinity evaluation were selected as described above.

## 3 Experimental

### 3.1 Materials used in the tests

*Dermatophagoides* mites (Biostir Inc., Osaka, Japan) and German cockroaches (SUMIKA TECHNOSERVICE CORPORATION, Hyogo, Japan) were used in the tests. As mentioned above, adding a surfactant to a pesticide will improve the ability of the preparation to wet the surface of the body of a pest to improve the efficiency with which the active ingredient permeates and kills the pest. We therefore measured the HSPs for the body parts of the pests.

Surfactants increased the efficiency with which the active ingredients penetrated the oil-protected German cockroach surfaces. Selecting an optimal surfactant required the HSPs for the different surfactants to be measured. The surfactants studied were dodecyl phosphate, hexadecyltrimethylammonium chloride, monomyristin, monopalmitin, monostearin, and trimethylstearyl ammonium bromide (Tokyo Chemical Industry, Tokyo, Japan).

### 3.2 Experimental methods

#### 3.2.1 Method for measuring the *Dermatophagoides* mite HSP

A 0.01 g aliquot of *Dermatophagoides* mites was placed in a glass tube, and 20 mL of an organic solvent was added. The sample was sonicated for 30 min and then shaken at room temperature for 24 h, then the affinity between the solvent and mites was evaluated visually. The criteria used to determine the affinity are described below. Poor and good solvents were identified from the affinity evaluation results, and the HSPs were determined using the Hansen solubility sphere method.

#### 3.2.2 Method for measuring the German cockroach HSP

A German cockroach was placed in a glass tube, and 50 mL of an organic solvent was added. The sample was shaken at room temperature for 24 h, then the affinity between the solvent and cockroach was evaluated visually. The criteria used to determine the affinity are described below. Poor and good solvents were identified from the affinity evaluation results, and the HSPs were determined using the Hansen solubility sphere method.

#### 3.2.3 Method for measuring the surfactant HSPs

A 0.01 g aliquot of a surfactant was placed in a glass tube, and 10 mL of an organic solvent was added. The sample was allowed to stand for 24 h, then the affinity between the solvent and surfactant was evaluated visually. The criteria used to determine the affinity are described below. Poor and good solvents were identified from the affinity evaluation results, and the HSPs were determined using the Hansen solubility sphere method.

## 4 Results and discussion

### 4.1 HSP results for *Dermatophagoides* mites

**Fig. 1:**
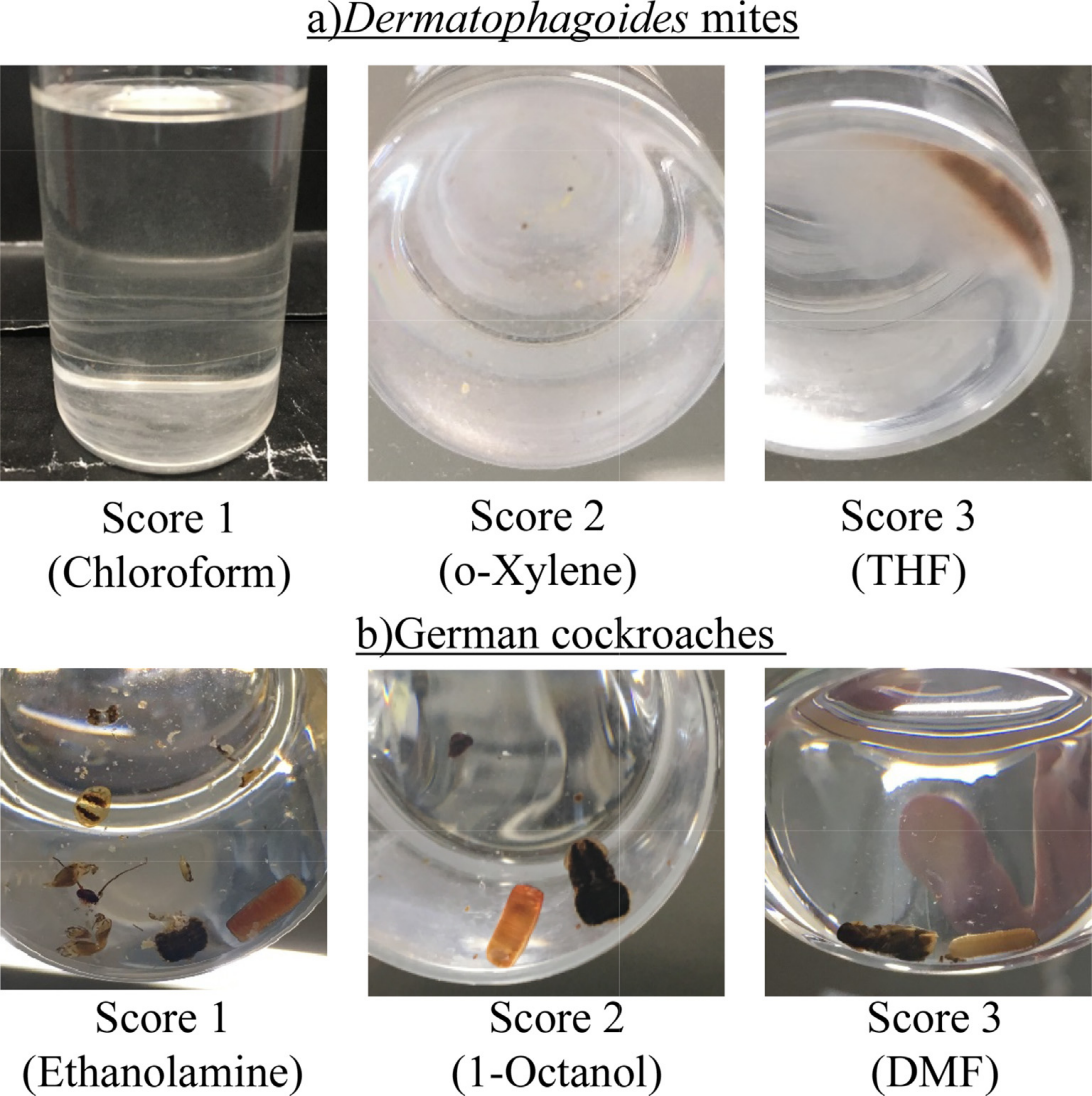
Examples of the affinity evaluation scores for a) *Dermatophagoides* mites: Score 1 was defined as they being dispersed and translucent, score 2 as they being mostly dispersed but somewhat precipitated at the bottom of the vessel, and score 3 as they being only precipitated at the bottom of the vessel. and b) German cockroaches: A score of 1 was defined as the nodes being destroyed and each part of the cockroach becoming separated, a score of 2 was defined as only the head becoming detached, and a score of 3 was defined as it not changing.

The affinities with various organic solvents were investigated. The evaluation criteria scores are shown in Fig. 1. Score 1 was defined as *Dermatophagoides* mites being dispersed and translucent, score 2 as *Dermatophagoides* mites being mostly dispersed but somewhat precipitated at the bottom of the vessel, and score 3 as *Dermatophagoides* mites being only precipitated at the bottom of the vessel. Good solvents in the Hansen solubility sphere method had scores of 1 or 2, which indicated that the affinity between the *Dermatophagoides* mites and the solvent was good. Poor solvents had a score of 3, which indicated that the affinity between the *Dermatophagoides* mites and solvent was poor.

**Fig. 2:**
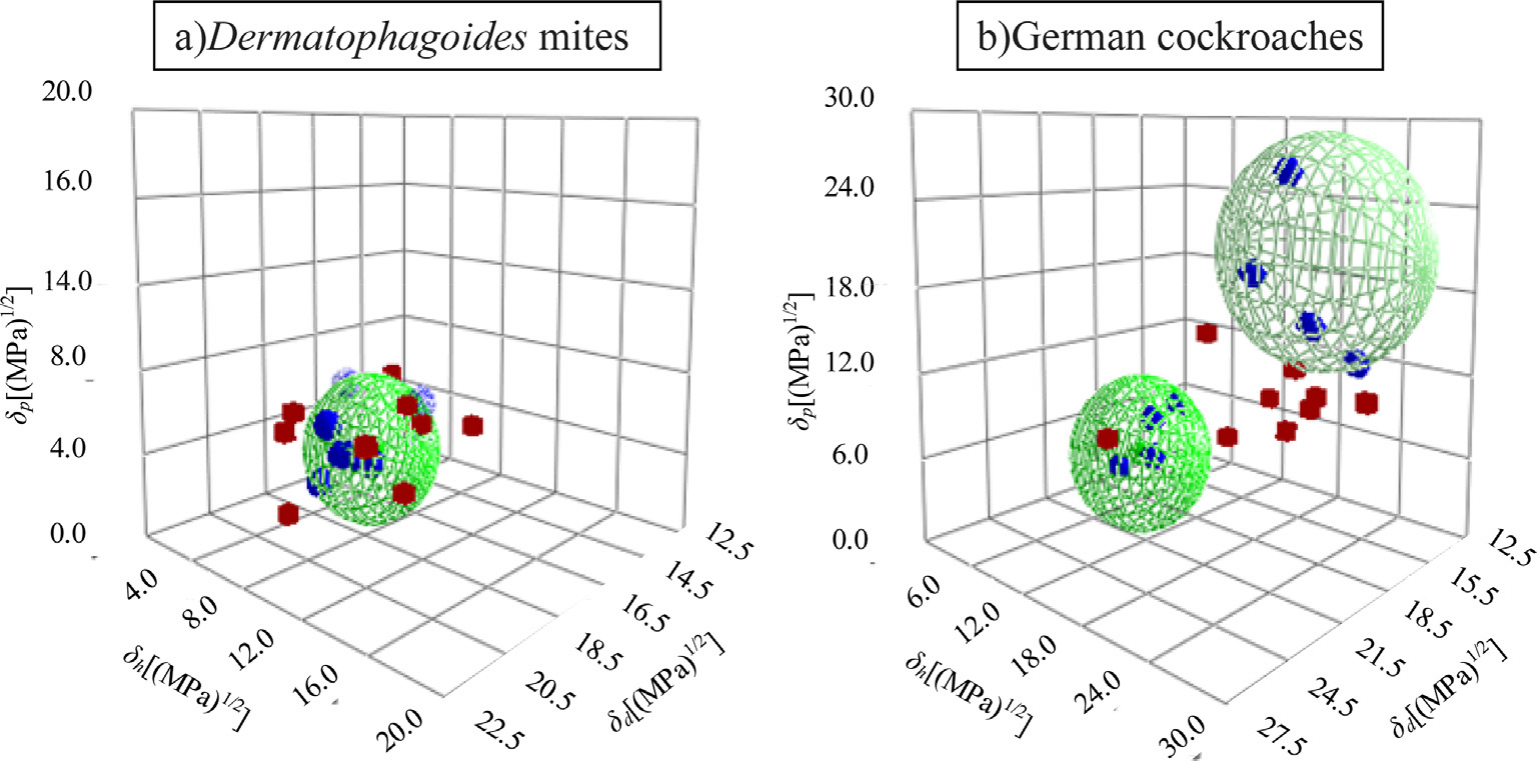
Hansen solubility spheres for a)*Dermatophagoides* mites and b)German cockroaches. German cockroaches had two types of sphere, one with a high *δh* and another with a low *δh*.

The Hansen solubility sphere results obtained using the HSPiP program are shown in Fig. 2. The *Dermatophagoides* mite HSPs were *δ*_*d*_ = 16.4 (MPa)^1/2^, *δ*_*p*_ = 2.6 (MPa)^1/2^, and *δ*_*h*_ = 4.7 (MPa)^1/2^.

Assuming that the four substances that were the main constituents of the mites (see section 2.2) were equivalent, we estimated the *Dermatophagoides* mite HSPs taking the volume fraction of each substance into consideration. The approximate values were *δ*_*d*_ = 17.1 (MPa)^1/2^, *δ*_*p*_ = 1.5 (MPa)^1/2^, and *δ*_*h*_ = 3.5 (MPa)^1/2^. The *R*_*a*_ values for the measured HSPs and estimated HSPs were 2.1 (MPa)^1/2^. The *R*_*a*_ values were <3.0 (MPa)^1/2^, and were assumed to indicate the same properties. The difference between each value was caused by the components used to estimate the value not being the same and because components other than the main component were affected in different ways.

### 4.2 HSP results for German cockroaches

The affinities with various organic solvents were investigated, and the evaluation criteria scores are shown in Fig. 1. A score of 1 was defined as the nodes being destroyed and each part of the cockroach becoming separated, a score of 2 was defined as only the head becoming detached, and a score of 3 was defined as the cockroach not changing. Good solvents in the Hansen solubility sphere method had a score of 1, which indicated that the solvent damaged the cockroach. Poor solvents had scores of 2 or 3, which indicated that the cockroach was almost unchanged by the solvent.

The Hansen solubility spheres determined from these results using the HSPiP program are shown in Fig. 2. German cockroaches had two types of sphere, one with a high *δ*_*h*_ and another with a low *δ*_*h*_. The HSPs of the high *δ*_*h*_ sphere were *δ*_*d*_ = 15.5 (MPa)^1/2^, *δ*_*p*_ = 20.4 (MPa)^1/2^, and *δ*_*h*_ = 20.2 (MPa)^1/2^, and the HSPs of the low *δ*_*h*_ sphere were *δ*_*d*_ = 17.6 (MPa)^1/2^, *δ*_*p*_ = 2.8 (MPa)^1/2^, and *δ*_*h*_ = 3.8 (MPa)^1/2^.

The HSPs of chitin (a constituent of the cockroaches) were *δ*_*d*_ = 17.7 (MPa)^1/2^, *δ*_*p*_ = 14.9 (MPa)^1/2^, and *δ*_*h*_ = 18.4 (MPa)^1/2^. The experimentally determined HSPs had relatively high *δ*_*p*_ and *δ*_*h*_ values, so we concluded that it was possible to make accurate measurements for the German cockroach surfaces. The *R*_*a*_ between the experimental and calculated results was 7.3 (MPa)^1/2^, meaning the measured *R*_*a*_ was probably different from the estimated *R*_*a*_. This would have been because the cockroach body contains other components (e.g., water).

### 4.3 Selection of solvents strongly compatible with *Dermatophagoides* mites

Efficiently exterminating mites requires a solvent with a good affinity for mites to be used. We identified mixed solvents with strong affinities for *Dermatophagoides* mites and low toxicities to humans. The optimum mixed solvent, a 52:48 v/v mixture of D-limonene and n-amyl acetate, had an *R*_*a*_ = 0.5 for *Dermatophagoides* mites. *D*-Limonene is found in citrus peel and is expected not to be toxic to humans. *n*-Amyl acetate is used in banana-type aromas, and is also not expected to be toxic to humans.

**Fig. 3:**
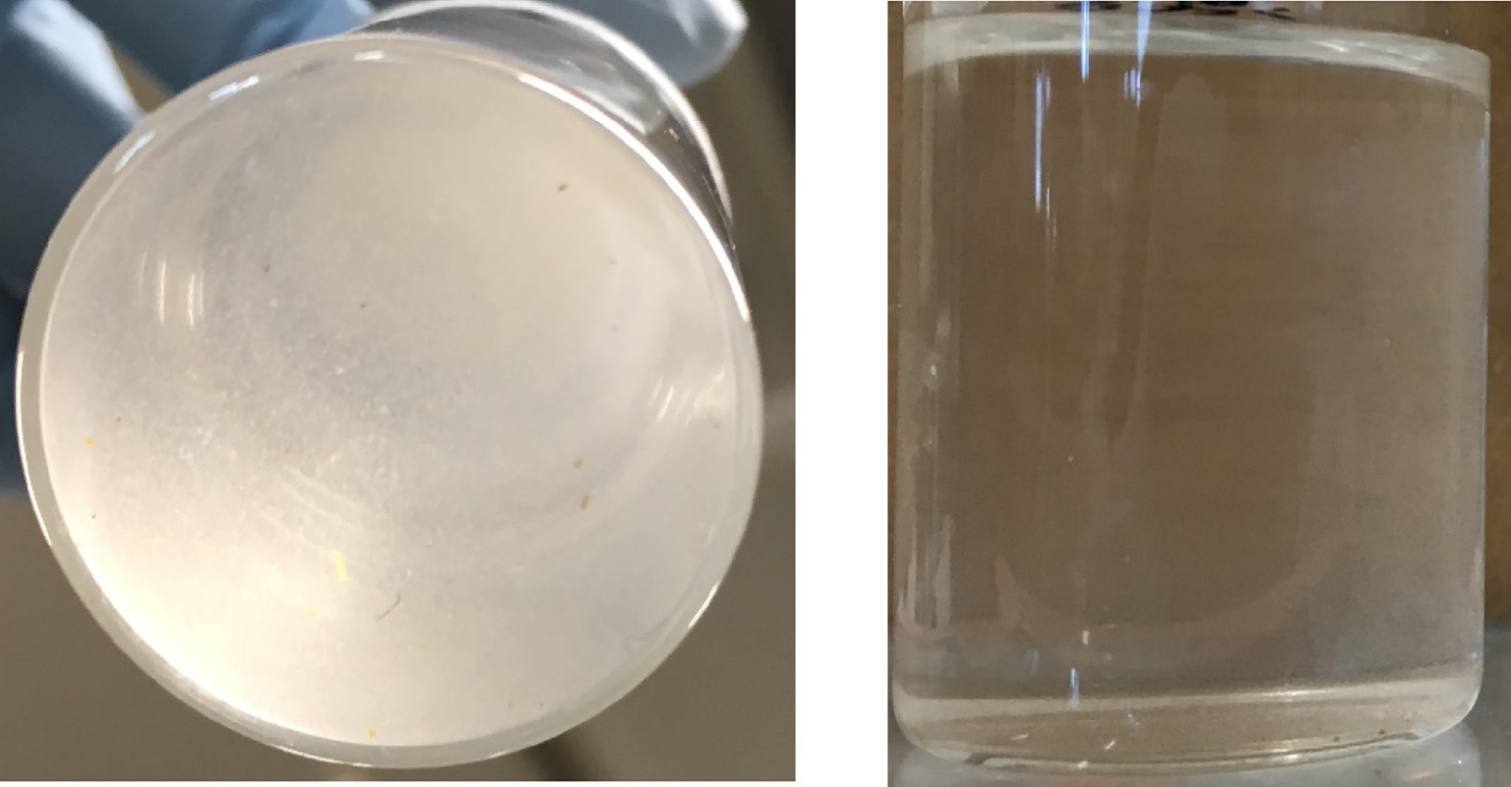
Results for The optimum mixed solvent, a 52:48 v/v mixture of D-limonene and n-amyl acetate, had an *Ra* = 0.5 for Dermatophagoides mites. They were dispersed and translucent.

The results for the optimum mixed solvent are shown in Fig. 3. The experiments were performed using the conditions described in section 3.2.1. *Dermatophagoides* mites became very dispersed and translucent in the optimum mixed solvent, so this solvent was given a score of 1. Mixing pure solvents can decrease the *R*_*a*_ values for *Dermatophagoides* mites and the solvent, and an optimum mixed solvent with a high affinity for *Dermatophagoides* mites was able to be designed. Such a mixed solvent should allow an optimum (safe and efficient) pesticide formulation to be designed.

### 4.4 Selection of a surfactant strongly compatible with the HSP measurements for the surfactants and German cockroaches

A surfactant with optimum ability to allow an insecticide to penetrate and control German cockroaches was identified. Surfactants have hydrophilic and hydrophobic properties, so have two types of HSP. The German cockroach surface is covered with oil, so German cockroach bodies also have two types of HSP. Therefore, when a surfactant solution is applied to a German cockroach, the HSPs on the hydrophobic side of the surfactant will most strongly affect the HSPs on the hydrophobic side of the cockroach. A surfactant that minimizes the *R*_*a*_ of the hydrophobic HSP of a surfactant and the HSP of the low *δ*_*h*_ sphere of the German cockroach should therefore be optimal. This time, six major surfactants were prepared as selection subjects. We compared the affinities between six common surfactants and German cockroaches.

The affinities with various organic solvents were evaluated. Good solvents in the Hansen solubility sphere method had scores of 1, which indicated that the surfactant dissolved well in the solvent. Poor solvents had a score of 2, which indicated that the surfactants poorly dissolved in the solvent.

The HSPs corresponding to the hydrophobic groups of the surfactants and HSPs of the cockroaches are shown in Table 1. It can be seen that the hydrophobic part of monostearin had the smallest *R*_*a*_ (4.3). This suggested that a solution containing monostearin (which had a low *R*_*a*_ and a good affinity for German cockroach surfaces) would allow a pesticide to permeate German cockroaches efficiently.

**Table 1:** *Ra* values for German cockroaches and hydrophilic Hansen solubility parameters for various surfactants

Sample	δ_d_ [(MPa)^1/2^]	δ_p_ [(MPa)^1/2^]	δ_h_ [(MPa)^1/2^]	R_a_ [(MPa)^1/2^]
Monostearin	16.8	2.5	7.8	4.3
Monopalmitin	17.5	5.9	8.7	5.8
Monomyristin	16.9	8.7	8.4	7.6
Hexadecyltrimethyl ammonium Chloride	17.3	9.5	13.9	8.0
Trimethylstearyl ammonium Bromide	17.4	7.7	10.3	8.1
Dodecyl phosphate	16.0	7.3	10	8.3

## 5 Conclusions

The affinities between various organic solvents and *Dermatophagoides* mites and German cockroaches were evaluated, and HSPs for *Dermatophagoides* mites and German cockroaches were calculated. Determining the HSPs allowed the surface properties to be quantitatively expressed. The *Dermatophagoides* mite surface components and body components are similar, so the dissolving sphere became a ball. The German cockroach had two types of dissolving spheres because the body contains highly polar components such as chitin and the surface is composed of low polarity oils. We succeeded in formulating an optimum mixed solvent taking the *Dermatophagoides* mite HSPs into account. It was possible to suggest a method for selecting a surfactant with a good compatibility with the German cockroach surfaces using the German cockroach HSPs.

The results suggest that the physical properties of *Dermatophagoides* mites and German cockroaches can be quantitatively expressed using HSPs and that the HSPs could allow the development of pesticide formulations that are more effective and less toxic to humans than currently available formulations.

## Declarations

### Author contribution statement

Yuki Kato: Conceived and designed the experiments; Performed the experiments; Analyzed and interpreted the data; Wrote the paper.

Tsutsumi Shinichi, Nobuyuki Fujiwara, Hideki Yamamoto: Analyzed and interpreted the data.

### Funding statement

This research did not receive any specific grant from funding agencies in the public, commercial, or not-for-profit sectors.

### Competing interest statement

The authors declare no conflict of interest.

### Additional information

No additional information is available for this paper.
